# Light Conscious Sedation in Patients with Previous Acute Myocardial Infarction Needing Exodontia: An Observational Study

**DOI:** 10.7759/cureus.6508

**Published:** 2019-12-30

**Authors:** Fabio Dell'Olio, Saverio Capodiferro, Pantaleo Lorusso, Luisa Limongelli, Angela Tempesta, Maria Massaro, Salvatore Grasso, Gianfranco Favia

**Affiliations:** 1 Interdisciplinary Department of Medicine, University of Bari Aldo Moro, Bari, ITA; 2 Emergency Department, University of Bari Aldo Moro, Bari, ITA

**Keywords:** myocardial infarction, community dentistry, risk assessment, conscious sedation, tooth extraction, oral surgery

## Abstract

Aim

This study evaluated a protocol of light conscious sedation for multimodal analgesia in patients with a clinical history of acute myocardial infarction requiring tooth extraction and to assess postoperative pain by using the quantity intake of acetaminophen as the indicator.

Material and methods

All 50 patients received preliminary anxiolysis via oral chlordemethyldiazepam administration. After 15 to 20 minutes, only patients reporting they were not fully relaxed received additional intravenous diazepam before tooth extraction. Acetaminophen 1000 mg was suggested as the preferred postoperative analgesic drug.

Results

The studied patients included 39 women and 11 men with a mean age of 69.4 ± 17.1 years. They were classified according to the American Society of Anesthesiologists Physical Status classification system as follows: 12 patients belonging to class II, 32 patient to class III, and the remaining six to class IV. Based on the Modified Dental Anxiety Scale, six patients were phobic and seven anxious. Nevertheless, intravenous conscious sedation was needed in 23 patients via diazepam. The first day after surgery, 76% of patients took acetaminophen, and 58% took acetaminophen the second day, with a mean two-day total intake of acetaminophen of 1020 ± 789 mg/day. Stratified statistical analysis was performed and revealed that 60.87% of patients receiving intravenous diazepam needed to take acetaminophen on the first day after dental extraction in contrast to the 88.89% of patients who did not receive intravenous diazepam (χ^2^ test; P = .021).

Conclusions

Our data suggest that anxiety related to dental procedures is widespread, although often unmentioned by patients. Moreover, the percentage of patients needing analgesics during the first 24 hours following surgery demonstrated that overall perioperative pain might be controlled by the light conscious sedation protocol for patients with previous acute myocardial infarction proposed in the current study.

## Introduction

Management of anxiety and pain is important for preventing stress-related cardiovascular accidents before, during, and after dental extractions in patients with a clinical history of acute myocardial infarction. Anxiety’s manifestations include cardiovascular reactions that may represent a source of stress for patients, such as vasovagal fainting characterized by an increase of heart rate and blood pressure at first with subsequent bradycardia and hypotension-the latter remaining the most common emergency in the dentist’s chair [1]. In fact, in such instances, anxiety activates the hypothalamus-pituitary-adrenal axis, inducing cortisol secretion and provides for endogenous catecholamine (mainly epinephrine) release over 10 times more than normal, evoking a strong cardiovascular response with blood pressure and heart rate augmentation, potentially straining a myocardium already weakened by past ischemia [1].

When anxiety is associated with aggression and anger, norepinephrine is released as well [2]. The total amount of circulating catecholamine also includes the exogenous levels injected into tissues during local infiltration of anesthesia. The latter may be involved in causing hemodynamic complications together with the endogenous peak; the true role of local anesthetic-related vasoconstrictors is still controversial [3]. Furthermore, some studies reported anxiety-related electrocardiogram (ECG) abnormalities such as ventricular premature contracture and atrial fibrillation that are present in 2.9% and 1% of patients, respectively, during local anesthesia execution by a general dentist [2].

Also, pain alerts the hypothalamus-pituitary-adrenal axis, increasing the release of both epinephrine and norepinephrine [2]. This is why inadequate analgesia in patients with previous acute myocardial infarction may represent a risk.

Anxious patients report more pain experienced than non-anxious ones, both during and after a surgical procedure [4]. This can be explained by a strong statistical correlation between the amount of anticipated pain, actual experienced pain, and total intake of anti-inflammatory drugs, with a direct proportion between them. The highest pain is generally reported in the first 24 hours after surgery [5]. There is also strong evidence for benzodiazepines (BDZ) as reducers of preoperative anxiety, experienced pain, and postoperative pain [5-6].

The current study aims to present a light conscious sedation protocol able to provide multimodal analgesia in patients with a history of acute myocardial infarction requiring tooth extraction and to assess postoperative pain using the quantity of acetaminophen intake as an indicator.

## Materials and methods

The authors studied 50 patients referred to the Complex Operating Unit of Odontostomatology, Aldo Moro University of Bari, needing teeth extracted from 2017 to 2018 and matching the following inclusion criteria: a clinical history of acute myocardial infarction occurring at least one year prior; ongoing antiplatelet therapy without any possible suspension for dental procedure; acetaminophen as a possible drug administrable as a postoperative analgesic; no allergic reactions to lidocaine, mepivacaine, diazepam, and acetaminophen; and negative anamnesis for neuropsychiatric disease. All patients provided informed consent to join the study.

A panoramic radiogram, standard blood tests, ECG, and an anesthesiological and cardiologic evaluation were performed for all patients. Each patient was classified according to the American Society of Anesthesiologists Physical Status (ASA PS) classification system, Modified Dental Anxiety Scale (MDAS), and Newman’s test [7-8]. For easier management of intra- and postoperative bleeding, extraction of only up to two teeth was suggested by a cardiologist. To stabilize cardiovascular parameters, chlordemethyldiazepam (CDDZ, En) drops in amounts varying from 0.5 mg to 1 mg were administered orally to patients with an MDAS score lower than 14, while anxious (MDAS ≥ 14) and phobic (MDAS ≥ 18) patients received 1 mg to 2 mg of CDDZ. Patient vital parameters were continuously monitored by a pulse oximeter, noninvasive blood pressure measurement every five minutes, and also by a three-lead continuous ECG system for the entire procedure. Patients reporting to be not fully relaxed after a waiting period of 15 to 20 minutes after CDDZ administration received additional intravenous diazepam using the following administration algorithm: an initial infusion of 2 mg followed by 1 mg every two minutes until reaching maximum individual tranquility (the end-point of sedation) or a maximum limit of 8 mg. Assessment of sedation was made one minute after each diazepam administration, by using an analogic scale ranging from 0 (absence of tranquility) to 10 (maximum individual tranquility) [9]. After achieving the end-point of sedation, tooth extractions were performed after topical anesthesia using a lidocaine 10 g/100 mL spray and injection of mepivacaine 3% (1.8 mL vials) without adrenaline. Patients underwent a postoperative Newman’s test to assess attention, motility, and cognitive level and were discharged one hour after the test was negative (omission of ≥ 7 points). Acetaminophen 1000 mg tablets (maximum of three/day) was suggested as postoperative analgesic therapy. We asked patients to record the total number of acetaminophen tablets taken in the first two days after surgery. This total was reported during a phone interview that would be performed during the third day or the one-week follow-up. The current study was designed to measure the actual pain experienced by patients via the consumption of acetaminophen to bypass visual analogical scale-related biases.

All data regarding age, sex, ASA PS classification, MDAS score, doses of CDDZ and diazepam, and acetaminophen tablets consumption during the first and the second day after surgery were collected for statistical analysis with a type I error estimated at 5% (P < .05).

The study was carried out in accordance with the principles of the Declaration of Helsinki. Patients provided informed consent for diagnostic and therapeutic procedures performed for research purposes.

## Results

Table 1 summarizes all the results obtained in the current study. The patients were 39 women and 11 men with a mean age of 69.4 years and a standard deviation (SD) of 17.1 years. The ASA PS classification of risk assessed 12 patients as ASA II, 32 as ASA III, and six as ASA IV. According to the MDAS score evaluation, six patients were phobic and seven anxious, while 37 had an MDAS score lower than 14. CDDZ was administered to all patients (0.8 mg ± 0.46 mg) while, despite MDAS scores, intravenous conscious sedation was needed in 23 patients instead of the 13 expected from the diazepam group (DG); the remaining individuals requiring this were from the no-diazepam-group (NDG). During the first day after surgery, 76% of patients took acetaminophen while 58% did during the second, giving a mean two-day total intake of acetaminophen of 1020 ± 789 mg/day. Stratifying patients between DG and NDG, we observed that DG received on average 1.022 mg of CDDZ (SD = 0.574 mg) while NDG received 0.611 mg (SD = 0.212 mg); no statistical difference was found between the two groups (t-test; P = .999). DG received an average quantity of 2.444 mg of diazepam (SD = 1.303 mg). We found that 88.89% of NDG needed acetaminophen in the first day after dental extraction, in contrast to 60.87% of DG (summarized in Table 2); this was a statistically significant difference (χ2 test; P = .021). During the second day, the proportion of acetaminophen use was statistically similar in both groups (χ2 test; P = .704). The mean two-day total intake of acetaminophen between groups was statistically similar (t-test; P = .471): DG took 978 mg/day (SD = 859 mg/day) while NDG took 1056 mg/day (SD = 738 mg/day). Hemorrhagic complications never occurred.

**Table 1:** Sample characteristics Sample characteristics: 37 patients had a low MDAS score but 14 of them required supplementary anxiolysis before surgery regardless. Abbreviations: ASA PS, American Society of Anesthesiologists Physical Status; F, female; M, male; MDAS, modified dental anxiety scale; SD, standard deviation.

Characteristic	Number
Sex (M/F)	11 / 39
Age (mean ± SD)	69.4 ± 17.1 years
ASA PS classification (II/III/IV)	12/32/4
MDAS score (< 14/14-17/≥ 18)	37/7/6
Diazepam/No Diazepam Group	23/27
Patients requiring analgesic (Day 1)	38 (76%)
Patients requiring analgesic (Day 2)	29 (58%)
Mean two-day acetaminophen intake (mean ± SD)	1020 ± 789 mg/day

**Table 2:** Acetaminophen intake between no-diazepam and diazepam group The proportion of patients that needed acetaminophen is reported for the first (Day 1) and the second (Day 2) day after surgery. The bottom line lists the daily mean intake of acetaminophen (mean ± SD) for an average patient in each group. Abbreviation: SD, standard deviation.

	No Diazepam Group	Diazepam Group	P-value
Day 1	88.89%	60.87%	.021
Day 2	60.87%	55.56%	.704
Mean	1056 ± 738 mg/day	978 ± 859 mg/day	.471

## Discussion

This is a single-center prospective study performed in accordance with the definition of conscious sedation given by the General Dental Council, which is “keep patient conscious, with his protective reflexes and able to respond to verbal commands” [10]. Furthermore, the administration of a BDZ with primary anxiolytic activity is advisable before dental procedures. Usually, diazepam and CDDZ are preferable to midazolam, as in the present study, as diazepam acts on the α2 subunit of the GABAA receptor, providing a strong anxiolytic effect and weak sedative effects, while midazolam targets the α1 subunit, thereby being strongly sedative and weakly anxiolytic [9,11]. Eventually, the α2 subunit is also involved in the prevention of hyperalgesia, providing a low analgesic effect, prolonged by the long half-life of diazepam, ranging between 30 and 56 hours [12]. This study shows that the percentage of patients of DG suffering very little in the first 24 hours from surgery and needing no rescue analgesic (acetaminophen) was about 20% more than those in the NDG group. This difference disappeared during the second day, mainly because some of NDG patients refused to take acetaminophen and instead waited for spontaneous reduction of pain. These different behaviors during the second day after surgery explain why the total consumption of acetaminophen was statistically similar between the two groups.

The combination of conscious sedation with the endpoint of maximum individual tranquility and correct local anesthesia provides a perioperative experience that allows safe treatment of at-risk patients. This study differs from other reports as it enrolled mainly patients with an ASA score ≥ III (76%). These patients have been treated in a fashion providing a continuous stabilization of vital parameters during the entire perioperative period.

## Conclusions

Our data suggest that anxiety related to dental procedures is widespread, though often unreported by patients. Perioperative pain may be controlled by the suggested light conscious sedation protocol in patients including those with previous acute myocardial infarction. Further studies could confirm the encouraging results obtained in this study possibly in randomized controlled trials to provide more evidence and better treatment of patients with myocardial ischemia.
